# Ecosystem Conditions That Influence the Viability of an Old-Forest Species with Limited Vagility: The Red Tree Vole

**DOI:** 10.3390/ani13071166

**Published:** 2023-03-25

**Authors:** William L. Gaines, Andrea L. Lyons, Lowell H. Suring, Carol S. Hughes

**Affiliations:** 1 Washington Conservation Science Institute, Leavenworth, WA 98826, USA; 2 Northern Ecologic L.L.C., Suring, WI 54174, USA; 3 Forest Service, Pacific Northwest Region, Portland, OR 97204, USA

**Keywords:** *Arborimus longicaudus*, ecosystem conditions, Bayesian networks, climate change, conservation, wildfire, habitat loss, old forests, timber harvest

## Abstract

**Simple Summary:**

Red tree voles (*Arborimus longicaudus*) occur throughout old forests in the Western United States of America. Ecosystem conditions needed to support the long-term viability of red tree voles have decreased throughout their range. This has primarily resulted from a reduction and modification of their habitats. We evaluated the risk of maintaining viable populations of red tree voles in three ecoregions within their range. We determined that the long-term probability of maintaining viability within these ecoregions ranged from 26% to 52% when compared to historical conditions. The most immediate threats varied from habitat loss due to timber harvest to habitat loss due to wildfires. Reducing the risks to the long-term viability of red tree voles will depend largely on the implementation of conservation practices designed to protect remaining habitat, restore degraded ecosystems, and adapt ecoregions to climate change.

**Abstract:**

We evaluated ecosystem conditions known to influence the viability of a strictly arboreal species (the red tree vole, *Arborimus longicaudus*) endemic and historically distributed in the forests across the Coast Range, Cascades, and Klamath Mountains ecoregions in the Western United States of America. We found widespread reductions in ecosystem conditions needed to support the long-term viability of the red tree vole. This was particularly evident in the Coast Range where the weighted watershed index (WWI) was 26% of its historical value, and the current probability of maintaining viability departed the most from historical viability probabilities in ecoregions that were evaluated. In contrast, in the Cascades and Klamath Mountains, the WWI was 42% and 52% of their respective historical values, and the current probabilities of maintaining viability departed less from historical conditions than in the Coast Range. Habitat loss from timber harvest represented the most immediate threat in the Coast Range, while habitat loss from wildfires represented the most risk to the red tree vole in the Cascades and Klamath Mountains. Reducing the risks to the viability of the red tree vole will depend largely on the implementation of conservation practices designed to protect remaining habitat and restore degraded ecosystems in the Coast Range. However, the risk of large, high-severity wildfires will require the protection and increased resilience of existing ecosystems. Our results indicate that considerable adaptation to climate change will be required to conserve the red tree vole in the long term. Conservation may be accomplished by revising land and resource management plans to include standards and guidelines relevant to red tree vole management and persistence, the identification of priority areas for conservation and restoration, and in assessing how management alternatives influence ecosystem resiliency and red tree vole viability.

## 1. Introduction

The red tree vole (*Arborimus longicaudus*) is endemic to Western Oregon and Northwestern California, and is one of two truly arboreal Arvicoline mammals in the world [1]. The red tree vole depends entirely on coniferous forests for its life history requirements, and they are among the few mammals that feed primarily on the needles and twigs of conifers [2,3,4]. They build nests on complex tree branch and bole structures, including broken tops, cavities, palmate branch whorls, large limbs, forked trunks, and dense limb structures that are near fresh conifer needles, which are also used for food [4,5,6]. Previous studies have shown that red tree voles are strongly associated with mature and old-forest (i.e., >80 years old) characteristics, such as complex branch and bole structures [7,8,9,10]. However, some researchers have also found red tree voles in young forest (i.e., 20–80 years) [5,11,12]. Occupancy in young forests may be positively influenced by the proximity to mature and old forests [6,10,12,13].

Forsman et al. [8] developed contemporary maps of red tree vole habitat using forest structure, forest composition, and abiotic (summer fog, temperature, precipitation) variables. They compared those maps to the historical maps of mature forest from 1914 to 1936 to estimate the amount of habitat change within the geographic distribution of the red tree vole in Oregon. They estimated the total extent of red tree vole habitat in 1914 to be 3.3 million ha, but by 1936, extensive timber harvest and the Tillamook Burn [14] had reduced habitat extent to 2.8 million ha. Finally, they estimated the extent in 2006 to be 1.2 million ha, which included habitat that had developed since 1914 from forest succession. This represented a 65% reduction in habitat extent between 1914 and 2006 [8]. Most (70%) of the remaining red tree vole habitat in 2006 occurred on federal lands. Linnell et al. [10] documented additional losses from recent (2017, 2018, 2020) high-severity fires on federal lands.

In addition to habitat extent, habitat patch size is important for species viability, especially for species with limited mobility. Red tree voles are short-distance dispersers (i.e., <75 m) [6,10,13]. Adults have been observed moving within distances ranging from 4 to 162 m ($\overset{-}{x}$ = 45 m) between alternate nests [6,12], while juveniles dispersed between 50–75 m to new nests [6]. Previous studies have identified habitat patches >20 ha as biologically relevant in supporting red tree vole occupancy [10]. For example, Linnell and Lesmeister [13] used >20 ha of old-forest patches as nodes in their analysis of red tree vole habitat connectivity. Forsman et al. [8] suggested that red tree voles may also use regenerating stands adjacent to mature or old forest, and remnant patches of old forest may also be important refugia or facilitate dispersal between larger patches of habitat [13].

The spatial dynamics of habitat patches can have considerable influence on species occupancy and persistence, especially for species with limited vagility, such as the red tree vole [12,13]. Forsman et al. [8] demonstrated that not only has the extent of habitat for tree voles been reduced, but the habitat that remained is also less contiguous and occurs in much smaller patches than historical conditions. In Oregon, they found a highly fragmented network of small patches within a matrix of predominately young forest. This resulted from a 98% reduction in the average patch size of potential habitat when comparing the habitat in 1914 to the habitat in 2006 [8].

The red tree vole was listed as a Survey and Manage Species with the adoption of the Northwest Forest Plan (NWFP) in 1994 [15]. This action required federal land management agencies to survey for and manage habitats for red tree voles (i.e., USDA Forest Service; USDI Bureau of Land Management (BLM)). As a result, the distribution, ecological relationships, and habitat requirements of the red tree vole were investigated in depth, e.g., [7,10,16,17]. The North Oregon Coast Distinct Population Segment (DPS) of red tree voles is a candidate for threatened or endangered federal designation [18,19] and is currently being re-assessed to determine its status and potential listing under the federal Endangered Species Act [20].

Units of National Forest System lands (i.e., National Forests) managed by USDA Forest Service are required to address the viability of species of conservation concern as forest plans are amended or revised [21,22]. The Pacific Northwest Region of the USDA Forest Service (Region 6) will be revising forest plans [23] under the 2012 planning rule [22]. Information with regard to the status of species of conservation concern will be critical in informing the planning process. Specifically, we conducted this species viability evaluation using the process described in Suring et al. [21] and Gaines et al. [24], with minor modifications, to assess the status of the red tree vole. We use the term “species viability evaluation” to reflect differences between the USDA Forest Service evaluations of viability and a formal population viability analysis [22,25]. Our approach to evaluating ecosystem conditions that influence viability may be broadly applicable to a wide-range of species which often lack detailed information needed to conduct formal population viability analyses.

Our evaluation focused on whether ecological conditions are likely to support a viable and persistent population of red tree voles over the long term under existing management plans [22]. The USDA Forest Service policy on conducting viability evaluations specifies that the following factors be considered: (1) species habitat needs and how they may be affected by management plan components; (2) trends in the quality, quantity, and distribution of habitat; and (3) trends in species abundance and distribution, to the extent these data are available [22]. We address all of these factors in our evaluation of the viability of the red tree vole. Our evaluation area included three ecoregions [26] within the range of the red tree vole in the Western USA: Coast Range, Cascades, and Klamath Mountains.

Specifically, our objectives in conducting this evaluation were to: (1) assess how the latest information on habitat relationships of red tree voles, characteristics of habitats, and habitat trends influenced viability, (2) assess how known risk factors currently influence viability, and (3) provide information to support forest plan revisions or amendments (e.g., as per the viability requirements of the most recent planning rule in effect in the USA [22]).

## 2. Materials and Methods

### 2.1. Study Area

**Figure 1:**
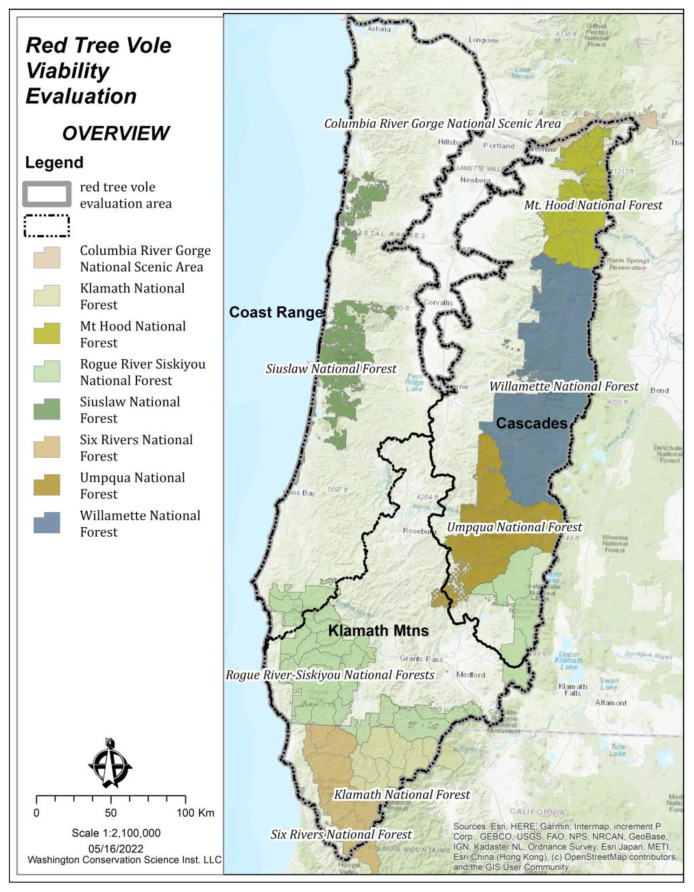
Evaluation area, ecoregions, and national forests located within the range of the red tree vole (*Arborimus longicaudus*) in Oregon and California, USA.

**Figure 2:**
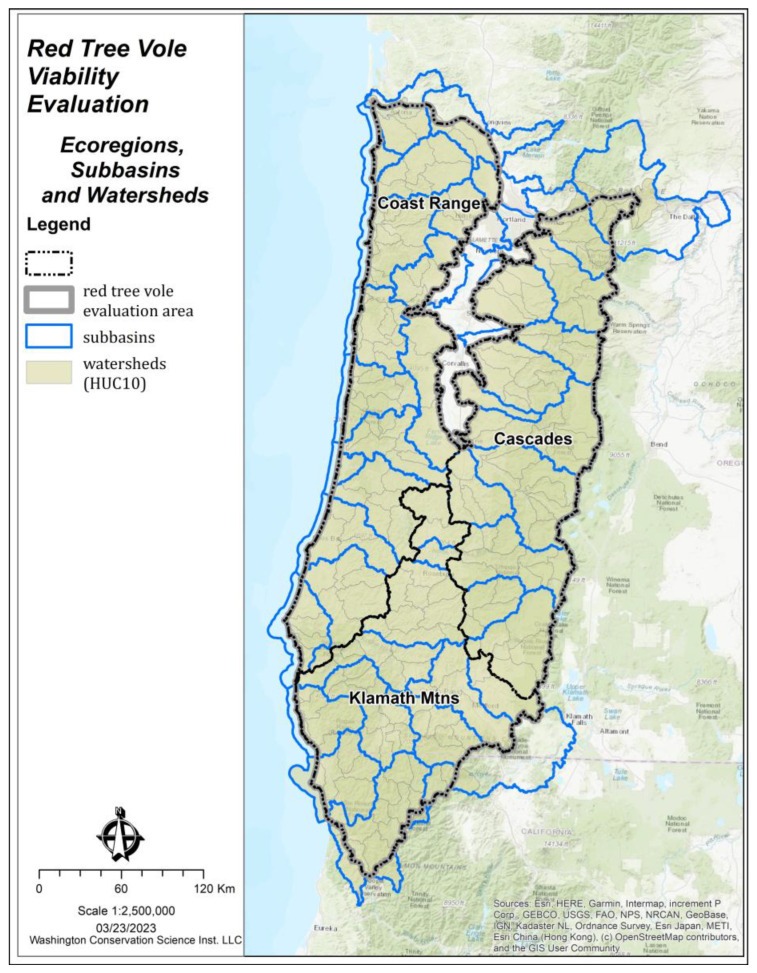
Watersheds, sub-basins, and ecoregions in the red tree vole (*Arborimus longicaudus*) evaluation area, Oregon and California, USA.

The evaluation area encompassed the historical range of the red tree vole [8,16] and was divided into three ecoregions (Coast Range, Cascades, and the Klamath Mountains [26]) to account for ecological differences and varying risk factors (Figure 1). These ecological differences included moisture regimes, tree species composition, and disturbance regimes. Our evaluation area included all watersheds (Hydrologic Unit Code [HUC] 10) and sub-basins (HUC 8) (i.e., medium-sized river basins) that overlapped our study area (Figure 2). Our evaluation area included 222 watersheds and 37 sub-basins. Watersheds in the Willamette Valley, Oregon, USA in the north-central portion of the evaluation area that did not contribute to red tree vole habitat were excluded from analyses. The watersheds ranged from ~75 to 1065 square kilometers in size and the sub-basins ranged from ~570 to 5590 square kilometers (Figure 2).

#### 2.1.1. Coast Range

Highly productive, rain-drenched, coniferous forests cover the low mountains of the Coast Range of Western Oregon and Northwestern California. Sitka spruce (*Picea sitchensis*) forests originally dominated the fog-shrouded coast, while a mosaic of Western redcedar (*Thuja plicata*), Western hemlock (*Tsuga heterophylla*), and Douglas-fir (*Pseudotsuga menziesii*) blanket inland areas. Currently, Douglas-fir plantations are prevalent on the intensively logged and managed landscape. In Oregon, soils are typically Inceptisols and Andisols, while Alfisols are common in the Californian portion of the ecoregion. The Coast Range consists of the least amount of land managed by federal agencies (24%) and the most amount of private land (63%), including large industrial forest areas. In addition, 12% is managed by the state of Oregon.

#### 2.1.2. Cascades

The Cascades ecoregion is a mountainous ecoregion that stretches through West-central Oregon. It is underlain by Cenozoic volcanic soils and much of the ecoregion is affected by alpine glaciation. In Oregon, the Western Cascade Mountains are dissected by numerous, steep-sided stream valleys. A high plateau occurs to the east, with both active and dormant volcanoes. The Cascades ecoregion has a moist, temperate climate that supports an extensive and highly productive coniferous forest that is intensively managed. At lower elevations in the north, Douglas-fir, Western hemlock, Western red cedar, big leaf maple (*Acer macrophyllum*), and red alder (*Alnus rubra*) are typical components of the forests. At higher elevations, Pacific silver fir (*Abies amabilis*), mountain hemlock (*Tsuga mertensiana*), subalpine fir (*Abies lasiocarpa*), noble fir (*Abies procera*), and lodgepole pine (*Pinus contorta*) occur. Subalpine meadows and rocky alpine zones occur at the highest elevations. Soils are mostly of cryic and frigid temperature regimes, with some mesic soils at low elevations. Andisols and Inceptisols are common. A considerable portion of the Cascades Ecoregion is under federal management (63%) and much of the remainder is in private ownership (35%).

#### 2.1.3. Klamath Mountains

The Klamath Mountains is a physically and biologically diverse ecoregion that covers the highly dissected ridges, foothills, and valleys of the Klamath and Siskiyou mountains. The region’s geology includes a mix of granitic, sedimentary, metamorphic, and extrusive rocks. It was unglaciated during the Pleistocene epoch, when it served as a refuge for Northern plant species. The region’s diverse flora, a mosaic of both Northern Californian and Pacific Northwestern conifers and hardwoods, is rich in endemic and relic species. The mild, subhumid climate of the Klamath Mountains is characterized by a lengthy summer drought. A considerable portion of the Klamath Mountains is under federal management (58%) and in private lands (40%), including industrial forest lands intermingled with federal lands.

### 2.2. Bayesian Networks

We developed Bayesian network (BN) models using the Netica^®^ modeling shell (Norsys Software Corporation, Vancouver, British Columbia, Canada) to provide a structured tool for the integration of several sources of information [21,24,27]. We used BNs to combine empirical data and information derived from expert knowledge to inform values of the variables and to assess the relationship and influence of those variables on the viability of the red tree vole [28,29,30,31]. The probabilistic nature of BNs allowed us to account for uncertainty associated with the variables that affect red tree vole viability.

We developed 2 models to evaluate red tree vole viability, similar to previous analyses [21,24,32]. The first calculation provided an index of habitat suitability for each watershed using factors known to influence red tree vole abundance, distribution, and demography. The second model estimated the probability of an ecoregion to support a sufficiently abundant and well-distributed population of the red tree vole [21]. At the ecoregion-scale we assessed factors known to influence viability outcomes for the red tree vole including habitat quality and abundance, habitat connectivity, and habitat distribution. Detailed descriptions of similar applications of BN models can be found in Suring et al. [21] and Gaines et al. [24].

### 2.3. Watershed Index Model

**Figure 3:**
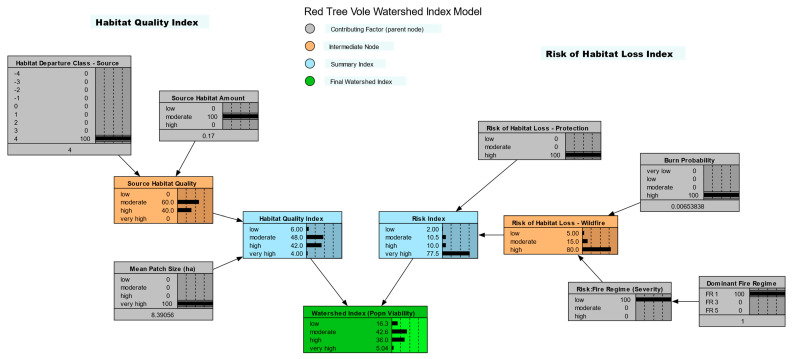
Red tree vole (*Arborimus longicaudus*) watershed index model for the evaluation area in Oregon and California, USA (with sample application).

The watershed index (WI) model (Figure 3) provided an index of the capability of a watershed to contribute to the viability of red tree voles [21,24]. Our selection of variables to include in the WI model was based on an extensive review of the literature, e.g., [5,6,7,8,9,10,11,12,13,16,17,19,33,34,35,36,37,38] and the expert review of our selections. The WI model outcome was estimated from spatial measures of habitat quality and risk factors (Figure 3). Habitat quality was based on the area of red tree vole habitat, the capability of the watershed to support habitat based on estimated historical conditions, and habitat patch size [21,24]. The risk portion of the WI model included potential habitat loss from timber harvest and wildfires [8,10,18,35,39].

### 2.4. Habitat Quality

#### 2.4.1. Source Habitat Amount

The source habitat we identified for red tree voles (Table 1) included characteristics of macro-vegetation that contributed to stationary or positive population growth and was distinguished from habitats that were simply associated with species occurrence [21,24,32].

**Figure 4:**
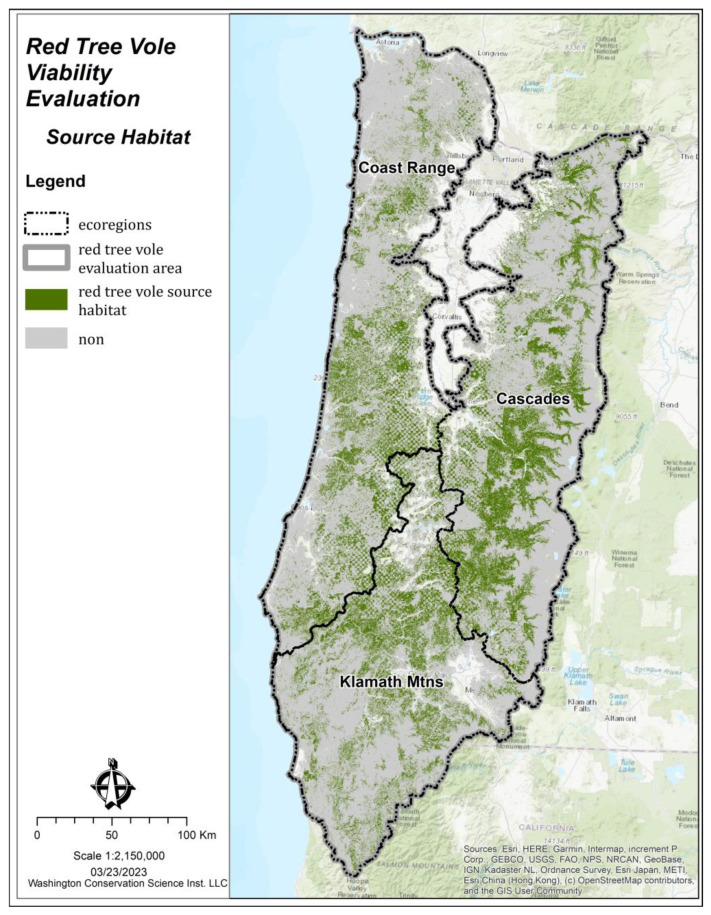
Source habitat for red tree voles (*Arborimus longicaudus*) by ecoregion within the evaluation area in Oregon and California, USA.

The area and spatial arrangement of source habitats (Figure 4) was summarized by the watersheds within each ecoregion (Figure 2). Within the WI model, the amount of source habitat was classified into three categories based on the Jenks Natural Breaks algorithm [40]: (1) low: watersheds with <15% source habitat, (2) moderate: watersheds with 15–30% source habitat, and (3) high: watersheds with >30% source habitat.

#### 2.4.2. Source Habitat Departure

We used estimates of the natural range of variability (NRV) for fire regime groups to estimate the potential of each watershed to provide red tree vole source habitat. NRV refers to the fluctuation in ecosystem structure, composition, and processes over time and is often used to understand the influence of anthropogenic change in ecological systems [41]. We used estimates of NRV derived from the literature [42,43,44,45,46] to determine how the area of current habitat changed in each watershed compared to the NRV, and to estimate departure from the NRV [21,24]. Departure refers to changes in extents and configurations of habitat that may have important ecological implications in terms of effects to species viability and persistence [21,47].

Habitat departure was assessed by calculating the ratio of current to potential habitat, and then comparing this ratio to the estimated NRV for each fire regime group (as described in Gaines et al. [24]). Fire regime groups provided a means of “binning” ecological variability based on differences in fire frequency and severity [48]. Potential habitat was identified using potential vegetation types (PVT) associated with red tree vole habitat and which have the potential to develop late-successional or old-forest structural stages in the absence of disturbance ([49], online resource 1). The habitat departure assessment provided a means of assessing the current and potential of each watershed to provide red tree vole source habitat. A key assumption was that the lesser the habitat departure, the greater the capability a watershed had for contributing to a positive viability outcome for the red tree vole.

#### 2.4.3. Habitat Patch Size

To assess the influence of habitat patch size, we used a habitat geospatial layer developed by Linnell et al. [38]. We used this to examine habitat patch size that approximated the biologically relevant patch size described by Linnell et al. [10] and used in Linnell and Lesmeister [13]. The mean habitat patch size within each watershed was classified into the following size classes: low (0–10 ha), moderate (>10–20 ha), high (>20–32 ha), and very high (>32 ha). An important assumption was that the greater the proportion of habitat in a watershed in larger patch sizes, the more capable the watershed was to contribute to the viability of red tree voles.

### 2.5. Risk Factors

There are several risk factors that may influence red tree vole viability (Table 2), the greatest of which is the conversion of old forest to young forest, primarily associated with timber harvest [8,10,35,39]. In addition, habitat loss from wildfire is increasing due to its interaction with climate change [8,10,35,38] and is a risk factor influenced by fire regime. Thus, we included variables that describe habitat loss related to timber harvest and wildfire.

#### 2.5.1. Status of Habitat Protection

Timber harvest influences the extent of red tree vole source habitat, with the harvest amount varying with land ownership and the level of habitat protection afforded by different management strategies. To quantify the level of protection for the existing source habitat within a watershed, we assigned three levels of protection based on land ownership and land management: protective (no timber harvest), restrictive (limited timber harvest), and non-protective (timber harvest with few restrictions). While these categories do not reflect a biological threshold, they are based on a key assumption that the higher the level of habitat protection, the better the watershed score and viability outcome. The level of red tree vole habitat protection was categorized as follows:

- high level of habitat protection (≥50% of source habitat in a watershed in the protective class),
- moderate level of habitat protection (≤60% of the source habitat in a watershed in the non-protective class and <50% in the protective class), and
- low level of habitat protection (>60% of the source habitat in a watershed in the non-protective class).

#### 2.5.2. Habitat Loss from Wildfire

The effects of past wildfires were reflected in the process for mapping source habitat [38]. The potential risk of future habitat loss due to wildfire was assessed by using data based on fire regime groups [46] and modeling burn probability from the Pacific Northwest quantitative risk assessment [50]. These data allowed an assessment of the risk to red tree vole habitat for both potential fire severity (i.e., fire regime group) and potential burn probability (i.e., how often a pixel was likely to receive fire).

Fire regime groups were characterized by likely fire behavior (i.e., burn severity and return interval) within landscapes based on interactions between vegetation dynamics, fire spread, fire effects, and spatial context [46]. The range of the red tree vole primarily comprised fire regime groups I (characterized by generally low-severity fires with some mixed-severity fires and fire-return intervals of 0–35 years), III (characterized by mixed-severity fires with some low-severity fires and return intervals of 36–200 years), and V (characterized by high-severity fires with return intervals of >200 years). Use of the fire regime groups allowed an assessment of potential fire severity and effects to red tree vole habitat. We used fire regime data from the LANDFIRE program, which provided a consistent dataset for the entire red tree vole evaluation area [49].

Burn probability was estimated for the entire red tree vole evaluation area as the likelihood of a pixel of habitat burning. We used a data layer generated from a comprehensive simulation system that used locally relevant fuel, climate, topography, and historical fire occurrence information to spatially estimate the likelihood and intensity of wildfire across the landscape [50,51,52]. The combination of fire regime and burn probability allowed us to assess the wildfire risk for each patch of habitat within a watershed. Our primary assumption in this assessment was that the lower the risk of habitat loss from wildfire, the better the WI score and the better the viability outcome for red tree voles.

**Table 2:** Summary of information on factors that pose risks or potential risks to the viability of red tree voles (*Arborimus longicaudus*) in the evaluation area in Oregon and California, USA.

Risk Factor	Comments	References
Habitat loss from timber harvest	Past harvest reflected in habitat maps; future harvest was indexed by landownership and management plans	[8,10,18,35,39]
Habitat loss from wildfire	Past fires reflected in current habitat maps; cumulative with effects from timber harvest; interacts with climate change; assessed by using fire regime (severity) and burn probability (frequency)	[8,10,18,35]
Predation—more information needed (not included in model)	Short-tailed weasel (*Mustela ermine*) and barred owl (*Strix varia*) may be exacerbated by forest management	[34,35]
Anticoagulant rodenticides (AR)—more information needed (not included model)	AR found in spotted and barred owls, even in remote parts of forest; indication of food web contamination	[53,54,55]

#### 2.5.3. Other Risk Factors

Other potential risk factors identified for red tree voles included increased predation (e.g., by short-tailed weasels (*Mustela erminea*) or barred owls (*Strix varia*)), exposure to rodenticides, and the effect on the habitat of Swiss Needle Cast (Table 2). Increases in predation may be influenced by habitat changes resulting from timber harvest [34,35]. Furthermore, agriculture, intensive forestry, and marijuana plantations may increase the risk of exposure of red tree voles to anticoagulant rodenticides [53,54,55]. However, these factors require further investigation and could not be quantified in the current assessment. The potential effect of Swiss Needle Cast on tree mortality is very limited in older forests and has only occurred in a small portion of the red tree vole range [56,57], so we did not include it as a risk factor in this assessment.

### 2.6. Sensitivity Analysis

Sensitivity analyses are useful for determining which habitat attributes might be prioritized for management in order to attain the greatest effectiveness in conservation or restoration planning. We performed sensitivity analyses in Netica^®^ to determine how much the values of a selected node in the watershed index model were influenced by a single finding at each of the other nodes. Sensitivity analyses in BNs evaluate the degree to which variation in the outcome (i.e., watershed index) is explained by other variables [30], identify the relative influence of each variable on the outcome, and can be conducted on any dependent node [58]. Variance reduction was calculated as the reduction in the variation of the value of the watershed index by each of the input variables using a routine in the Netica^®^ shell. The results were used to quantitatively compare and rank the effect of input variables on the model outcome.

### 2.7. Model Assessment

We assessed WI model performance by comparing WI scores with red tree vole occurrence data (similar to [21,25]). Occurrence data were obtained from unpublished federal agency databases and included detections of red tree voles, nests, or signs of red tree voles from 1982 to 2020 (N = 16,443). We tested the hypothesis that the mean WI values from red tree vole occurrence points would be greater than the mean WI values generated from an equal number of random points. We used two-sample *t*-tests for unequal variances with α = 0.05 to compare these values.

### 2.8. Viability Outcome Model

**Figure 5:**
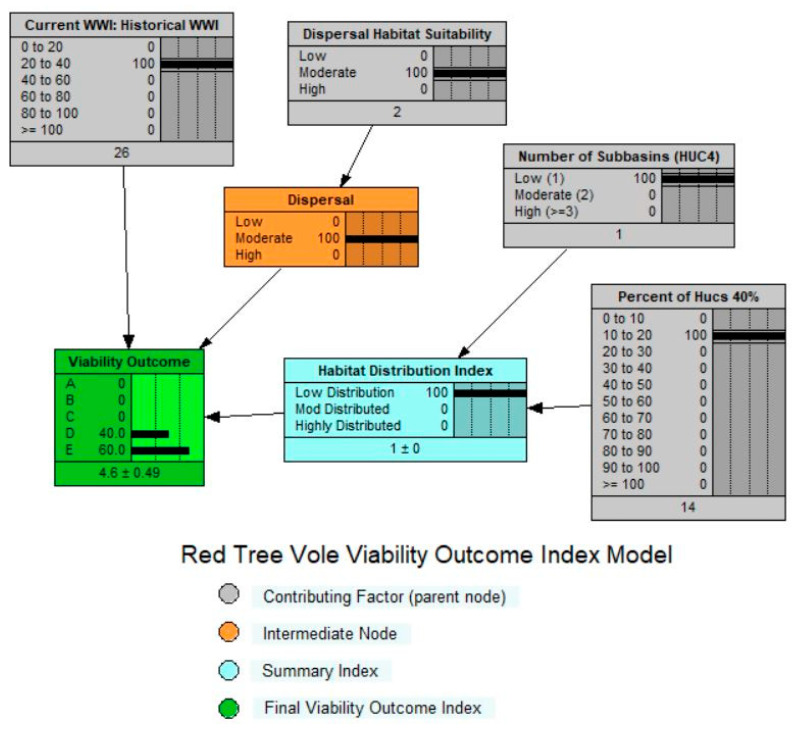
Red tree vole (*Arborimus longicaudus*) viability outcome index model for the evaluation area in Oregon and California, USA (with sample application).

The viability outcome model incorporated a WI score for each watershed, which we weighted by the habitat extent in the watershed, a distribution index for source habitats across the ecoregion, and a habitat connectivity index (Figure 5). While the viability outcome is a large-scale index of potential population abundance and distribution based on ecosystem conditions (habitat and risk factors) across an ecoregion, it is not an accurate prediction of population occurrence, size, density, or other demographic characteristics [21,24]. A key assumption was that locations with high viability outcomes would have a high probability of sustaining viable and well-distributed red tree vole populations in the evaluation area [21,24]. There were three primary components of the viability outcome model: (1) weighted watershed index, (2) dispersal habitat suitability, and (3) habitat distribution.

### 2.9. Weighted Watershed Index

The weighted watershed index (WWI) provided a measure of the capability of an ecoregion to contribute to the viability of red tree voles by comparing current conditions to the estimated NRV [24]. We calculated the WWIs (see [24] (p. 28)) and the ratio of the current WWI to the historical WWI within each ecoregion.

### 2.10. Dispersal Suitability

We assessed structural landscape connectivity, defined as the degree to which a watershed may facilitate or impede the movement of organisms [59]. We used a metric referred to as “dispersal suitability” [24], which is based on the concept that resistance to movement across a landscape can be mapped by assigning resistance values to land cover attributes [13,24,59]. Resistance surface mapping was developed by Linnell and Lesmeister [13] for a portion of the red tree vole’s range in the Northern Oregon Coast Range based on habitat suitability [10]. We extended this concept to create a resistance surface map for the whole red tree vole evaluation area.

We considered, among other elements, how human factors (e.g., major human travel routes, human development) may influence red tree vole movements. However, these factors were not encountered in the Linnell and Lesmeister [13] study area. Empirical studies indicating how these human factors influence the movements of red tree voles were not available. To identify these factors and assign resistance values, we relied on: (1) expert panels that convened to address red tree vole viability as part of the NWFP Survey and Manage Program, and (2) the literature on other small mammal species of similar size and habitat requirements (e.g., Townsend’s chipmunk (*Tamias townsendii*), Douglas squirrel (*Tamiasciurus douglasii*), Northern flying squirrel (*Glaucomys sabrinus*)). Expert panels had hypothesized that general forest roads would not likely limit tree vole movements, but that major highways and human developments would. Our review of the literature also suggested that local, low-use roads limited, but often did not prevent, crossing by small mammals, and that paved roads with heavy traffic often deterred the movement of small animals [60,61,62]. 

We used resistance values for major roads and human developments that were similar to those reported for the Northern flying squirrel, a similar arboreal mammal with limited dispersal ability [63]. We assigned resistance values that ranged from 0 (low cost of movement) to 500 (high cost of movement, Table 3). These resistance values were used in the Gnarly Landscape Utilities Resistance and Habitat Calculator [64] to develop a score for each pixel from low resistance (high dispersal suitability) to high resistance (low dispersal suitability). This resulted in a map that depicted the hypothesized “energetic costs” for a red tree vole to move across the landscape and was expressed as “Dispersal Suitability” [21,24].

We assessed the dispersal suitability score for an ecoregion by classifying the percentage of the ecoregion that was classified as high (resistance value ≤ 30), moderate (resistance value > 30 to 250), and low (resistance value ≥ 250). We then compared the current and estimated historical dispersal suitability. To estimate these historical dispersal conditions, we “turned off” the geospatial impact of major travel routes and human population density. We accounted for the effects that historical fire regimes may have had on dispersal habitat by assigning areas in fire regime groups III and V (in which fire frequency was historically low) as “high” dispersal suitability, and areas in fire regime group I (in which fire frequency was historically high) as “moderate” dispersal suitability. This allowed us to quantify the degree to which the potential for red tree voles to disperse across watersheds in the ecoregions had been influenced by human activities, and to identify areas (i.e., watersheds) where dispersal suitability was high and may be important to conserve, or areas where dispersal suitability was low but could be enhanced through restoration (e.g., forest management) or mitigation (e.g., highway crossing structures).

### 2.11. Habitat Distribution Index

The habitat distribution index was used to assess the spatial distribution of watersheds with relatively high amounts of source habitat within an ecoregion. The habitat distribution index was estimated by calculating the interaction of two variables: (1) number of sub-basins with at least one watershed that met a minimum threshold for the amount of source habitat (to assess how the “good” watersheds are distributed across the ecoregion) and (2) the percentage of watersheds at or above the threshold for source habitat. The threshold amount of source habitat within a watershed was assumed to be ≥40% of the estimated historical median of source habitat [21] (see [24] (p. 17) for further explanation). We categorized the percentage of watersheds within an ecoregion that met the 40% threshold into 10 equal categories from 0 to 100% (10% increments). The median value was calculated across all watersheds in an ecoregion.

We also estimated the habitat distribution index for historical conditions by determining which of the watersheds had historical estimates of source habitat amounts that were ≥40% of the median of the historical amount across all watersheds. We then used those watersheds to calculate the number of sub-basins with at least one watershed above the 40% threshold, and the percentage of watersheds with source habitat above the 40% threshold.

The habitat distribution index for both current and historical conditions was categorized as follows: (1) low (≤30% of the sub-basins with at least one watershed >40%); (2) moderate (>30–60% of the sub-basins with at least one watershed >40%), and (3) high habitat distribution (>60% of the sub-basins with at least one watershed >40%).

### 2.12. Viability Outcome

We developed the viability outcome model to assess ecosystem conditions for the red tree vole, and expressed them as a probability of having one or more of five viability outcomes (Table 4). The term “suitable environment” used in the viability outcomes referred to a combination of source habitat and risk factors that influence the probability of occupancy and demographic performance of the red tree vole based on our current understanding of their habitat and population relationships.

## 3. Results

### 3.1. Watershed Index

**Figure 6:**
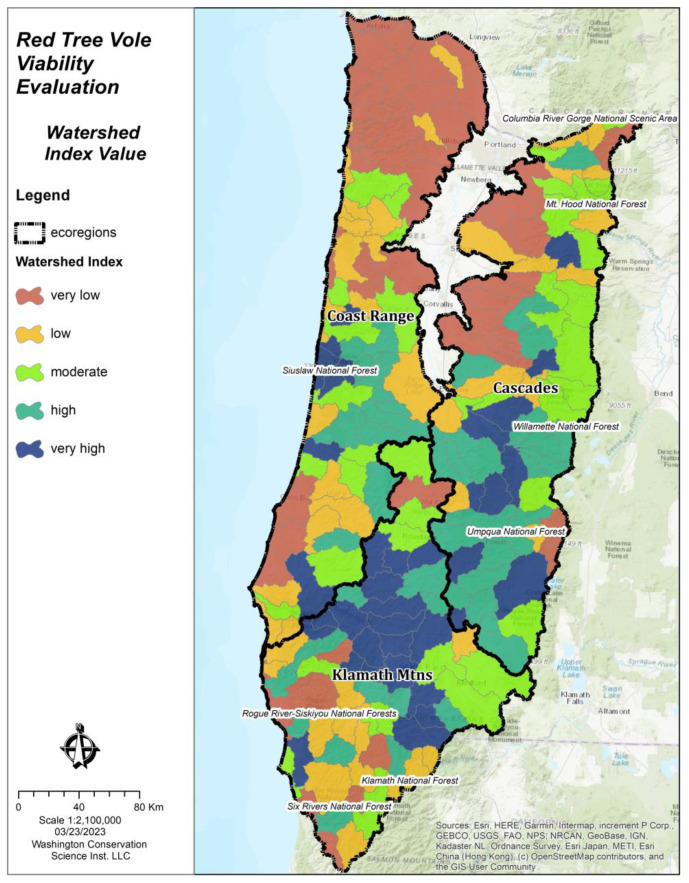
Watershed Index values by watershed in each ecoregion within the red tree vole (*Arborimus longicaudus*) evaluation area in Oregon and California, USA.

We assessed 222 watersheds (HUC 10) across the three ecoregions in the evaluation area (Table 5, Figure 6). We found that the current WI scores were lowest for the Coast Range ecoregion ($\overset{-}{x}$ = 0.49, N = 80), followed by the Cascades ecoregion ($\overset{-}{x}$ = 0.80, N = 77), and the highest in the Klamath Mountains ecoregion ($\overset{-}{x}$ = 1.01, N = 65). We found considerable departures in current WI scores compared to historical estimates, with the greatest departure in the Coast Range ecoregion. We also found that the historical WI scores were relatively lower in the Klamath Mountains ecoregion compared to the other ecoregions, likely due to the historical fire regime which limited the old forests that comprised source habitat for red tree voles [65].

We structured the WI model based on the assumption that the WI scores should be most influenced by source habitat variables, which included habitat amount, habitat departure, and mean patch sizes (Figure 3). The relative sensitivity analysis confirmed that the structure of the WI model supported this assumption. The rank of the relative sensitivity of watershed index values to variables in the Bayesian network model for red tree vole was characterized as follows: (1) amount of source habitat, (2) habitat departure class, (3) mean patch size, (4) level of protection, and (5) fire.

### 3.2. Watershed Index Model Evaluation

**Figure 7:**
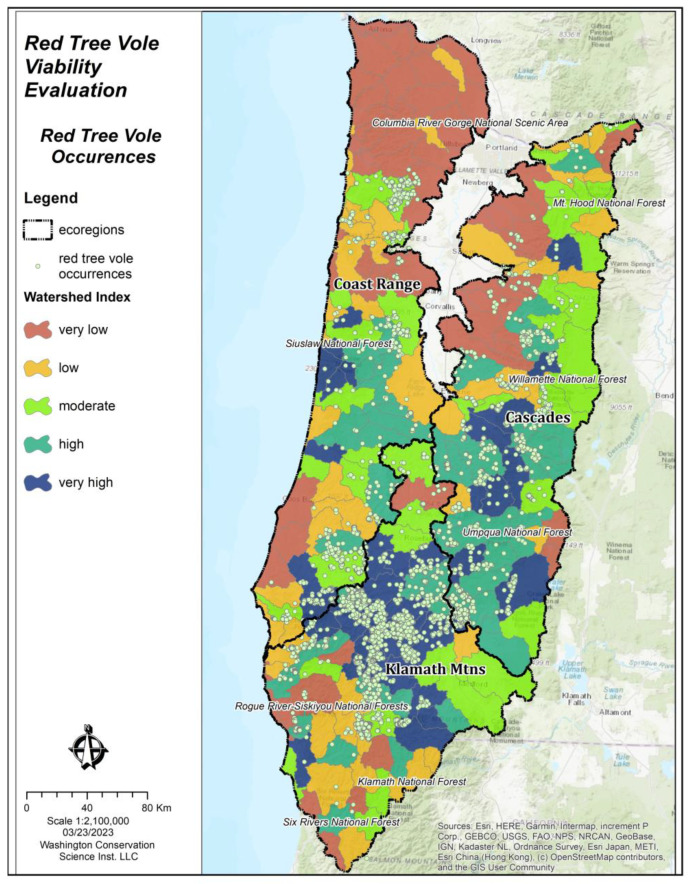
Red tree vole (*Arborimus longicaudus*) occurrences and watershed index values across ecoregions in the evaluation areas of Oregon and California, USA.

Results from the application of the WI model showed statistically significant (*t* = 69.0, *p* ≤ 0.001) support for the hypothesis that the mean WI values generated from red tree vole occurrence points (WI mean = 1.37, N = 16,433) was greater than those generated from random points (WI mean = 0.75, N = 16,433). There was a strong relationship between the WI value and the distribution of red tree vole occurrences (Figure 7), which indicated that our modeling approach was suitable for identifying the ecological conditions that influenced the occurrence of red tree voles.

### 3.3. Viability Outcomes

#### 3.3.1. Coast Range Ecoregion

The WWI scores indicated that the current habitat capability for red tree voles within the Coast Range ecoregion was 26% of its historical capability. Our evaluation showed that 18% of the Coast Range ecoregion had low dispersal suitability, 66% had moderate dispersal suitability, and 16% was high suitability.

**Figure 8:**
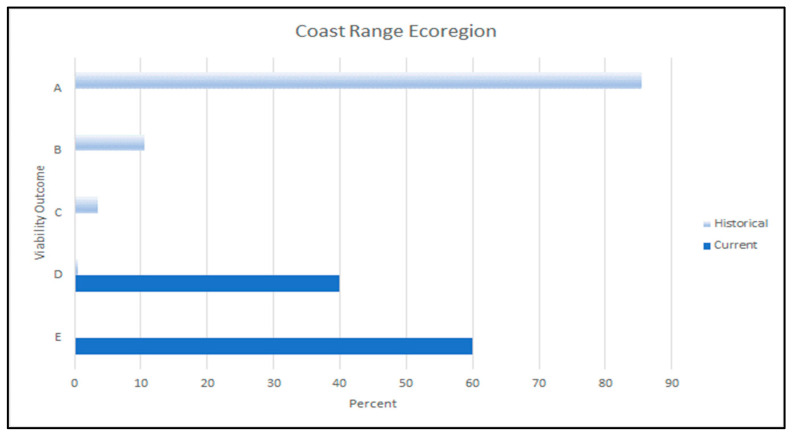
Historical (light blue bars) and current (dark blue bars) viability outcomes for red tree voles (*Arborimus longicaudus*) in the Coast Range ecoregion in Oregon, USA. Viability outcomes are described in Table 4.

The primary viability outcome for the red tree vole in the Coast Range was ‘E’ (Figure 8), indicating suitable environments (e.g., clusters of watersheds with high WIs) may be isolated or are low-to-moderately distributed across the historical range of the red tree vole within this ecoregion. In addition, relative to historical conditions, there were few suitable environments which were well-distributed within the Coast Range ecoregion. While some of the subpopulations associated with these environments may be self-sustaining, there was limited opportunity for populations to interact among many of the other suitable environmental patches, given the red tree vole’s limited dispersal ability. These conditions have likely resulted in a reduction in species’ range and the red tree vole may not currently be broadly distributed across the ecoregion. Among suitable environments, there was likely a low probability of interaction between populations, thereby increasing the potential for extirpations within these isolated areas.

Historically, when red tree vole habitat was more abundant and widely distributed in this ecoregion, dispersal would have been less limited than it currently is (Figure 8). We estimated that 90% of the sub-basins within the Coast Range ecoregion contained at least one watershed with >40% of the median amount of source habitat. Ninety-five percent of watersheds had >40% of the median amount of source habitat. Based on these estimated historical conditions, the viability outcome was primarily A, indicating that there was a high probability that viable populations of red tree voles were broadly distributed throughout the Coast Range ecoregion.

Of the habitat currently remaining in the ecoregion, 62% occurred on federal lands, of which 6% was not protected, 11% is in a restricted level of protection, and 83% is in the highest level of protection. The watersheds with the highest WI scores occurred in areas with substantial amounts of federal lands. However, the viability outcome for the Coast Range assessment area was still primarily outcomes D and E (Figure 8) due to the fragmented nature of the habitat that is largely a result of fragmented ownership patterns.

#### 3.3.2. Cascades Ecoregion

The WWI scores indicated that the current habitat capability for red tree vole within the Cascades ecoregion was 42% of the historical capability. As with the other ecoregions, there has been a considerable reduction in dispersal suitability and in the distribution of suitable environments. Our results showed that 24% of the Cascades ecoregion had low dispersal suitability, 63% had moderate dispersal suitability, and 13% had high dispersal suitability. Fifty percent of the sub-basins within the Cascades ecoregion contained at least one watershed with >40% of the median amount of historical source habitat. Twenty-five percent of watersheds had >40% of the median amount of historical source habitat. 

**Figure 9:**
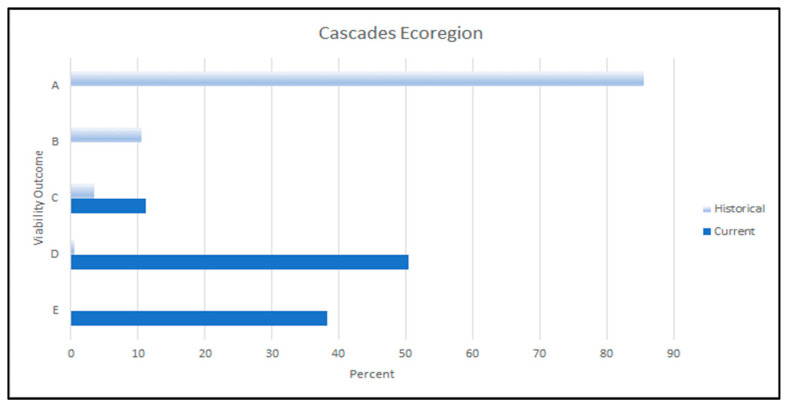
Historical (light blue bars) and current (dark blue bars) viability outcomes for red tree voles (*Arborimus longicaudus*) in the Cascades ecoregion in Oregon, USA. Viability outcomes are described in Table 4.

Our evaluation showed that the primary viability outcome under current conditions for the Cascades ecoregion is outcome D (Figure 9), indicating that suitable environments for red tree vole were relatively better in terms of their number and distribution when compared to the Coast Range ecoregion. In the Cascades ecoregion, suitable environments were moderately distributed across the red tree vole range and of moderate number relative to historical conditions. Where suitable environments were absent or present in low abundance, some subpopulations of red tree vole may have been isolated, particularly given their limited dispersal ability. A reduction in the species’ range in the Cascades ecoregion may have occurred and resulted in the red tree vole being broadly distributed in only a portion of the ecoregion.

Historically, suitable dispersal and source habitats would have been more widely distributed for red tree vole in the Cascades ecoregion when compared to current conditions. For example, we estimated that all fourteen of the sub-basins within the Cascades ecoregion contained at least one watershed with >40% of the median amount of historical source habitat and 96% of watersheds had >40% of the median amount of historical source habitat. Based on these conditions, the primary historical viability outcome was likely outcome A, similar to the Coast Range ecoregion (Figure 8). Under historical conditions, there was a high probability that viable populations of red tree voles were more broadly distributed when compared to current conditions throughout the Cascades ecoregion.

Among the three ecoregions that we assessed, the contribution of National Forest System lands to the conservation of red tree voles was greatest in the Cascades ecoregion (83%, of which 4% is not protected, 44% is in a restricted level of protection, and 52% has a high level of protection). The distribution of WI scores also showed that many of the highest values occurred on National Forest System lands.

#### 3.3.3. Klamath Mountains Ecoregion

The WWI scores indicated that the current habitat capability for red tree vole within the Klamath Mountains ecoregion was considerably reduced from its historical capability (i.e., 52% of the historical capability). However, the dominant historical fire regime in this ecoregion may have limited the historical abundance of red tree vole source habitats.

**Figure 10:**
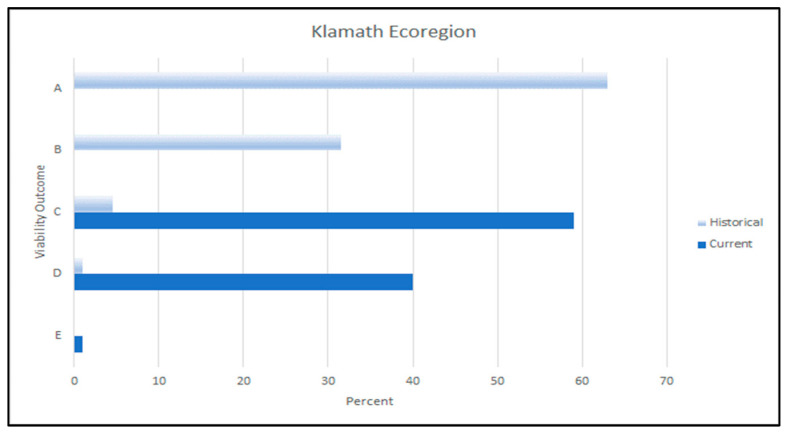
Historical (light blue bar) and current (dark blue bar) viability outcomes for red tree voles (*Arborimus longicaudus*) in the Klamath Mountains ecoregion in Oregon and California, USA. Viability outcomes are described in Table 4.

Dispersal conditions within the Klamath Mountains ecoregion were similar to those in the Coast Range ecoregion: 20% of Klamath Mountains had low dispersal suitability, 69% had moderate dispersal suitability, and 11% had high suitability. In the Klamath Mountains ecoregion, 85% of the sub-basins contained at least one watershed with >40% of the median amount of historical source habitat, and 84% of the watersheds had >40% of the median amount of historical source habitat. Based on this evaluation, the Klamath Mountains ecoregion had a primary viability outcome of C (Figure 10), indicating that suitable environments for red tree voles in this ecoregion were moderately distributed with moderate abundance relative to historical conditions. Gaps where suitable environments were either absent or present in low abundance were large enough that some subpopulations of red tree voles may have been isolated, given their limited dispersal ability. There may have been a reduction in the species’ range in the ecoregion and the red tree vole was likely broadly distributed in only a portion of the Klamath Mountains ecoregion.

Historically, suitable dispersal and source habitats would have been more abundant and better distributed than they are currently. For example, we estimated that all 13 of the sub-basins within the Klamath Mountains ecoregion contained at least one watershed with >40% of the median amount of historical source habitat and 97% of watersheds had >40% of the median amount of historical source habitat. 

Our estimates of the historical viability outcomes demonstrate that they were relatively lower in the Klamath Mountains ecoregion than the other two assessment areas. We noted that in the Klamath Mountains ecoregion the primary historical viability outcome was outcome A but there was a considerable probability of outcome B (Figure 10). This was likely due to the influence of the historical fire regimes (more frequent fires) on habitat amounts and distribution compared to the other ecoregions (less frequent fires). Under historical conditions, there was a moderate–high probability that ecosystem conditions to support viable populations of red tree vole were broadly distributed throughout the Klamath Mountains ecoregion. About 70% of the current red tree vole habitat occurred on federal lands within the ecoregion (of which 3% were in a non-protected status, 44% in restricted status, and 53% in the highest level of protection).

## 4. Discussion

We evaluated the ecosystem conditions that, based on our current understanding, are most likely to influence the viability of the red tree vole. Our WI model accurately identified watersheds with the highest likelihood of supporting red tree voles. In addition, the areas we identified as having the most suitable environments for red tree voles coincided with the results from several other assessments [7,8,10,18,38]. The process we used to evaluate ecosystem conditions known to influence the viability of red tree voles in Western Oregon and Northwest California, USA, may be readily applied to other species of conservation concern to assess their viability risk and to provide information to guide the management of their habitats. This approach may be broadly applicable as the vast majority of species lack detailed demographic and resource selection information necessary to conduct formal species viability analyses.

### 4.1. Coast Range Ecoregion

Similar to other studies, our evaluation of the conditions that influenced viability outcomes for red tree voles in the Coast Range ecoregion showed a low abundance and isolation of suitable environments, indicating a considerable reduction in the current range of the red tree vole compared to historical conditions. Forsman et al. [8] showed that substantial reductions in tree vole habitat occurred between 1914 and 2006 in the north, central, and southern portions of the Coast Range as a result of the extensive harvest of older forests and past fires. They also reported that the area in the northern most portion of the Coast Range had the greatest reduction in tree vole habitat, contributing to a ~80% contraction of the range of red tree vole [8]. Similarly, Linnell et al. [10] reported that old forest covering over the northern half of the Coast Range was reduced by >80% from 1911 to 2015. Finally, our findings support those of the USFWS [18] that the North Oregon Coast DPS of the red tree vole is in danger of extinction in the foreseeable future (i.e., within 60 years). USFWS determined that older forest habitats are limited and highly fragmented, and existing regulatory mechanisms on private and state lands are limited in their effectiveness to conserve populations of red tree voles [18].

In addition to the reduction in habitat extent from historical conditions in the Coast Range ecoregion, habitat connectivity had declined and fragmentation had increased [13]. Our models documented considerable reductions in dispersal suitability in this evaluation area, which were a result of timber harvest, human development, past wildfires, and major travel routes. Similarly, Forsman et al. [8] found a 98% reduction in the average patch size of potential tree vole habitat between 1914 and 2006. These conditions have resulted in a dramatic shift from a landscape dominated by suitable red tree vole environments to a highly fragmented series of small patches of habitat within a mosaic of young forests [8].

An important consideration for red tree vole conservation in the Coast Range is the role that fire will play in affecting the few remaining large patches of habitat. Highly clustered large forest patches provide important habitat for species in old forests, such as red tree voles. However, these patches are also susceptible to large, severe, stochastic events, such as wildfires [18]. The ability of these habitat conditions to absorb these events has been greatly reduced [13,18]. In addition, the effect of this risk factor is likely to increase considerably over time as climate change results in increases in the frequency of large, high severity fires [19,66]. 

In summary, our results showed that the viability of red tree voles within the Coast Range ecoregion had been reduced considerably from historical conditions. The reduction in distribution, number, and connectivity of suitable environments for red tree voles likely dramatically reduced their distribution and resulted in local extirpations [8,10]. In 2019, the US Department of Interior Fish and Wildlife Service completed a species status assessment for the North Oregon Coast DPS of the red tree vole [19]. That assessment concluded that this DPS of red tree voles has lost viability over the past 100 years due to habitat loss, resulting in a decline or extirpation of red tree voles in this area. Red tree voles currently occur in fragmented and isolated clusters primarily restricted to federal and state lands [19]. In addition, risk factors, including timber harvest, will continue to affect this species’ viability, and the effects of continued climate change will increase the threat of wildfires [18,66]. 

### 4.2. Cascades Ecoregion

Our results indicated that conditions to support red tree vole populations in the Cascades assessment area have been reduced from historical conditions, although not to the same degree as in the Coast Range ecoregion. The conditions that influenced red tree vole viability showed that suitable environments were low to moderately distributed across the ecoregion. These results suggest a likely reduction in the range of the red tree vole from historical conditions. This was particularly evident in the northern portion of the assessment area where habitat reductions have been the greatest. In addition, current suitable environments for red tree voles were well-distributed only in the southern portion of the Cascades ecoregion. Our results are corroborated by other assessments of habitat conditions for red tree voles in the Cascades. In the northern portion of the Cascades, Forsman et al. [8] documented a 71% reduction in red tree vole habitat and an estimated 73% reduction in their range between 1914 and 2006. In the remainder of the Cascades, Forsman et al. [8] reported a 52% reduction in red tree vole habitat between 1914 and 2006.

The cumulative effects of timber harvest and more recent fires have influenced the availability, distribution, and connectivity of habitats for the red tree vole. For example, during the summer of 2020, five large fires burned nearly 344,000 ha, some in watersheds with the best watershed index scores, reducing red tree vole habitat by nearly 67,180 ha within the perimeters of the fires [38]. Additional habitat is likely to be lost as a result of delayed mortality. In addition, this trend is likely to continue as a result of climate change. Davis et al. [66] showed that the area suitable for large fires will increase from 1% to 13–18% by the year 2100. Clearly, the risk factor associated with the loss of habitat to fires will increase considerably as temperatures increase and the fire season lengthens [66,67].

A sizeable portion (63%) of the Cascades ecoregion is under federal management, primarily the National Forest System lands. Our evaluation of the suitable environments that likely influenced the viability of the red tree vole found that the viability outcome for the northern portion of the Cascades ecoregion primarily had outcomes D and E, which likely reflected past habitat loss and the resulting reduction in the range of the red tree vole (as described above). The remainder of the ecoregion primarily had outcome C, which likely reflected the large concentrations of suitable environments with high WI scores. Due to the high percentage of federal lands in the Cascade ecoregion, forest management, including fire management, on these lands will be a primary driver influencing the viability outcome for red tree voles.

### 4.3. Klamath Mountains Ecoregion

Our evaluation of the conditions that influenced viability outcomes for the red tree vole in the Klamath Mountains ecoregion showed that suitable environments were moderately distributed across their historical range. Suitable environments currently exist at moderate abundance, primarily in the north central portion of the ecoregion. However, there were gaps in the suitable environments that may limit species distribution relative to historical conditions. Our results are corroborated by Forsman et al. [8] who found a 68% reduction in habitat for red tree voles from 1914 to 2006 in the Klamath Mountains ecoregion. 

Historically, wildfire has been an important factor in determining the availability and distribution of habitats associated with old forests within the Klamath Mountains ecoregion [65,68]. Wildfire will continue to be a major factor influencing the sustainability and resiliency of these old forests [38] due to the predicted impacts of warmer temperatures resulting from climate change [69]. Davis et al. [66] estimated that the area suitable for large fires would increase from 18 to 48–51% by the year 2100. Their estimate represents a considerable increase in fire and therefore the greatest risk to red tree vole viability, within the species’ current distribution.

The primary viability outcomes for the Klamath Mountains ecoregion were outcomes C and D (i.e., low to moderate distribution of suitable habitats, Table 4). The watersheds with the highest concentration of suitable environments occurred on the BLM-managed lands in the north-central portion of the ecoregion. In 1994, BLM management plans were amended by the Northwest Forest Plan [15], requiring that Survey and Manage Species, such as the red tree vole, be protected when required surveys resulted in detections of this species. In addition, habitats associated with old forests were protected in a series of late-successional reserves and riparian reserves that provided for the viability of old-forest species, such as the red tree vole [70]. In 2016, the BLM revised their resource management plans, and subsequently, the Survey and Manage standards and guidelines were not carried forward [71]. As a result, land use allocations were revised and the level of protection within some reserves was reduced (e.g., from protected to restricted) to allow more flexibility in implementing treatments to reduce fire risk. These treatments could be designed to reduce the risk of habitat loss from fires if planned at a landscape scale and if treatments and habitats are strategically located [72,73,74,75]. Forest restoration treatments planned at landscape scales could be applied to restore ecological processes [76], and increase forest heterogeneity (e.g., intermixing different forest types and/or age classes) that could enhance forest resistance and resilience [77]. In addition to their greater resilience, heterogeneous forest landscapes, within which old-forest habitat are embedded, frequently support a wide range of ecosystem functions and services [78].

## 5. Conclusions

Forest managers are faced with a challenge of how to create more resilient forests and landscapes while also conserving species diversity [79]. This is particularly challenging when dealing with a species whose habitat includes complex forest structures that can be difficult to sustain when faced with increasing risks of megafires [80,81]. Climate change is having an immediate and considerable impact on the habitat and viability of the red tree vole. In 2021, an additional 88,500 ha was burned in wildfires within our assessment area, 38,300 ha of which occurred in areas that we identified as source habitats. We suggest that the restoration of landscape resiliency in the face of climate change be given high priority as land management plans are revised. By using a landscape-level evaluation to strategically determine where to manage for habitats associated with old forest, using topographic features as a guide in this planning, and being strategic in the placement of restoration treatments to alter fire behavior, both resiliency and species conservation objectives may be achieved [79,80]. Fortunately, patch sizes of high quality red tree vole habitat can be relatively small (>20 ha) and still have considerable conservation benefits [10,38]. As we have demonstrated, the goals for the size and number of habitat patches may be informed by using historical and future reference conditions, and be integrated with goals of restoring pyro- and successional diversity [80]. Habitat connectivity between patches could be accommodated in two ways: (1) riparian reserves to provide corridors between habitat patches, and (2) softening the “matrix” by retaining small patches of old forest and perpetuating large, old tree structure in treatment units [82]. It appears that red tree voles may use younger forests (40–80 years old) that retain these structures [11,12].

The species viability evaluation process that we used [21,24] does not quantify extinction risk as is performed when using population viability analyses e.g., [83]. However, we expect that the poorer the viability outcome in our evaluation, the greater the risk of extinction. In addition, our approach relied on habitat monitoring, which is a valuable conservation tool and can be considerably less expensive than population monitoring. Ideally, however, some level of population monitoring should be applied to evaluate some key assumptions made in habitat-only assessments [38,84].

Our approach also produced information that may be used to inform the development and implementation of management strategies. For example, in the Coast Range, the development of a multi-ownership conservation strategy focused on the protection and restoration of old forests in strategically important locations could enhance the viability of red tree voles. This strategy could include retaining small patches of habitat that provide connectivity, as well as a retention of legacy structures (e.g., large trees) in treatment units [8,10,13]. Linnell and Lesmeister [13] identified key areas for the conservation or restoration of habitat connectivity. As we recommended, the areas they identified included a series of small, spatially central patches, and several large patches. Up to 30% of the highest priority areas were forested areas outside of existing reserves [13].

In the Cascades, strengthening existing protections on federally managed lands, identifying and protecting existing movement corridors, addressing the increasing threat of large wildfires, and restoring habitats in areas where suitable environments are of low abundance (e.g., the northern portion) would likely improve viability outcomes. Franklin and Johnson [85] recommended that all existing old forests on federal lands be protected as a means of strengthening federal provisions for providing ecosystem services. In the Cascades, forests have experienced an excess (higher than NRV) of large high-severity fire patches resulting from past forest management and ongoing climate impacts [65,80]. Restoration of mixed severity fire regimes would include a patchwork of successional stages, including late-successional forests that provide habitat for red tree voles, restore pyro-diversity, and enhance resiliency of late-successional forests (see Hessburg et al. [80]). Additionally, diverse silvicultural treatments could be applied in existing young forest stands to accelerate the development of structural complexity that are key elements of red tree vole habitat [80,85].

Finally, in the Klamath Mountains Ecoregion, where the risk of habitat loss from wildfire was the greatest, a management strategy that enhances protections of existing habitats on federal lands while actively managing to reduce wildfire risk would benefit red tree voles (see Jones et al. [79]). This could be accomplished by using forest restoration principles applied to landscape- and stand-scales, which would likely improve viability outcomes [72,86]. Such an approach would need to carefully consider the trade-offs associated with habitat loss from wildfires versus the effects of forest treatments on habitat suitability. A key component of such a strategy is the protection and enhancement of large and old trees [72,79,80].

Our evaluation also surfaced the need for further research and monitoring to address the conservation of red tree voles. For example, concerns about the effects of anticoagulant rodenticides and altered predator–prey interactions have been expressed [18,53,54,55] and need further research. Additionally, a better understanding of factors that influence red tree vole movements could resolve the assumptions we made in assessing quality of dispersal habitat, such as the influence of roads, highways, and large patches of high severity fire. It would be beneficial to better understand how different fire severities, and post-fire recovery, influence tree vole habitat use and dispersal. Learning more about the influence that restoration treatments have on red tree vole habitat use and habitat quality would aid in designing strategies to reduce the risk of habitat loss from fire versus effects to habitat loss from forest treatments [79,80]. We recognized that fire risk is likely to be considerably influenced by climate change [87,88], and this will need to be re-assessed in future iterations of this viability evaluation.

Our red tree vole viability evaluation may be used to inform revisions of federal land and resource management plans [22,89]. In general, watersheds with high concentrations of suitable environments could be prioritized for conservation, while watersheds with moderate scores may be the focus of management treatments that restore habitat quality, quantity, and connectivity. Our evaluation results could be used to update standards and guidelines relevant to red tree vole conservation and for assessing how management alternatives would provide for the ecological conditions that have the greatest influence on red tree vole viability [21,22,24]. Finally, continually updating red tree vole habitat maps will be vital in monitoring how wildfires and climate change influence habitat abundance and distribution, which are key factors that influence the viability of red tree voles [38].

## Figures and Tables

**Table 1:** Summary of information available to define and map habitat for red tree voles (*Arborimus longicaudus*) in the evaluation area in Oregon and California, USA.

Habitat Quality	Notes	References
**Source Habitat**—late-successional forest >80 years and old forest >200 years in Douglas-fir and Sitka Spruce zones.	Roughly analogous to suitable and highly suitable classes in Forsman et al. [8]	[7,8,9,10,33,36,37]
**Low Suitability Area**—forests 40–80 years of age even with patches of remnant older forest (>80 years) interspersed in Douglas-fir and Sitka Spruce zones.	Roughly analogous to marginal and unsuitable habitat class in Forsman et al. [8]	[8,10,34,37]

**Table 3:** Habitat variables and resistance values used to evaluate dispersal habitat suitability for red tree voles (*Arborimus. longicaudus*) in the evaluation area in Oregon and California, USA.

Habitat Variables	Resistance Values
Habitat Suitability:	
Not habitat but with potential to develop habitat	100
Marginal	50
Suitable	1
Highly suitable	0
Not habitat—not capable	500
Transportation Routes:	
Road (major or secondary highway)	500
Housing Density (dwelling units/ha):	
No buildings	1
Isolated buildings (0–3 buildings/0.4 ha)	5
Clusters of buildings (3–15 buildings/0.4 ha)	50
High density buildings (>15 buildings/0.4 ha)	100

**Table 4:** Viability outcomes used to describe amount, quality, connectivity, and distribution of current and historical suitable environments that influenced the viability of the red tree vole (*Arborimus longicaudus*) in the evaluation area in Oregon and California, USA.

Viability Outcomes—We used five viability outcomes that were calculated for current and historical conditions to assess changes in habitat conditions. The term “suitable environment” used in the viability outcomes refers to a combination of source habitat and risk factors that influence the probability of occupancy and demographic performance of the red tree vole based on our current understanding of their habitat and population relationships.
Outcome A—Suitable environments are broadly distributed across the historical range of the red tree vole throughout the ecoregion. Suitable environments are in high abundance relative to historical conditions. The combination of distribution and abundance of environmental conditions provides opportunity for continuous or nearly continuous intraspecific interactions for the red tree vole.
Outcome B—Suitable environments are broadly distributed across the historical range of the red tree vole. Suitable environments are of moderate to high abundance relative to historical conditions, but there may be gaps where suitable environments are absent or present in low abundance. However, any disjunct areas of suitable environments are typically large enough and close enough to permit dispersal among subpopulations and to allow the red tree vole to potentially interact as a metapopulation. Ecoregions with this outcome have suitable environments for red tree voles that are likely well-distributed throughout most of the area.
Outcome C—Suitable environments are moderately distributed across the historical range of the red tree vole. Suitable environments are moderately abundant relative to historical conditions. Gaps exist where suitable environments are either absent or present in low abundance, and given their limited dispersal ability, some subpopulations of red tree vole may be isolated. There may be a reduction in the range of the red tree vole in the ecoregion. Ecoregions with this outcome have suitable environments for red tree voles that are likely well-distributed in only a portion of the area.
Outcome D—Suitable environments are low to moderately distributed across the historical range of the red tree vole. Suitable environments exist at low abundance relative to their historical conditions. While some of the subpopulations associated with these environments may be self-sustaining, there is limited opportunity for population interactions among many of the suitable environmental patches for red tree voles given their limited dispersal ability. There may be a reduction in the range of the red tree vole in the ecoregion. Suitable environments for the red tree vole may not be well-distributed across the ecoregion.
Outcome E—Suitable environments are highly isolated and exist at very low abundance relative to historical conditions. Suitable environments are not well-distributed across the historical range of the red tree vole. There may be little or no possibility of population interactions among suitable environmental patches, resulting in potential for extirpations within many of the patches, and little likelihood of recolonization of such patches. There has likely been a reduction in the range of the red tree vole from historical conditions. Ecoregions with this outcome have suitable environments for the red tree vole that are not well-distributed throughout much of the area.

**Table 5:** Summary of the watershed index scores by ecoregion for red tree vole (*Arborimus longicaudus*) in the evaluation area in Oregon and California, USA.

		Watershed Index Scores			
Ecoregion	Number of Watersheds	Current		Historical	
		Mean	Range	Mean	Range
Coast Range	80	0.49	0.14–2.02	2.77	2.73–2.79
Cascades	77	0.80	0.13–2.82	2.76	2.73–2.79
Klamath Mountains	65	1.01	0.12–2.78	2.73	2.73–2.79

## Data Availability

The authors confirm that the data supporting the findings of this study are available within the article. Raw data supporting the conclusions of this article are available from the authors upon reasonable request.
